# Pathological Roles of Astrocytes in Traumatic Brain Injury

**DOI:** 10.1002/cns.70856

**Published:** 2026-04-12

**Authors:** Di Wu, Yuxiao Ma, Baofeng Wang, Yongtao Zheng, Jie Ren, Qingfang Sun, Liuguan Bian, Yuhao Sun

**Affiliations:** ^1^ Department of Neurosurgery, Ruijin Hospital Shanghai Jiao Tong University School of Medicine Shanghai P. R. China

**Keywords:** astrocytes, blood–brain barrier disruption, ion channel dysregulation, neuroinflammation, oxidative stress, reactive astrogliosis, traumatic brain injury

## Abstract

**Background:**

Astrocytes participate in both neuropathological and protective processes following traumatic brain injury (TBI) and undergo various characteristic changes, including phenotypic transformation, transcriptional reprogramming, and functional diversification.

**Methods:**

A comprehensive literature review was conducted in PubMed using key terms “astrocytes” and “traumatic brain injury”, and we integrated this existing evidence from transcriptomic analyses to mechanistic studies to elucidate the role and underlying mechanisms of astrocytes in TBI pathophysiology.

**Results:**

Transcriptomic analyses reveal distinct astrocyte phenotypes: neurotoxic A1 astrocytes and neuroprotective A2 astrocytes. Beyond this binary framework, single‐cell studies have identified intermediate astrocyte states, underscoring the need for more nuanced functional profiling. TBI triggers astrocyte activation via classic signaling pathways in response to mechanical stress, damage‐associated molecular patterns (DAMPs), and cytokines. These pathways—TLR4/NF‐κB, JAK/STAT3, and MAPK—form an interactive signaling network, enabling astrocytes to integrate diverse injury signals into coordinated responses that drive subsequent pathological effects. Dysregulation of astrocytic ion channels and transporters disrupts ionic homeostasis, exacerbating cytotoxic and vasogenic edema. Mitochondrial dysfunction and reactive oxygen species overproduction further amplify neuronal damage through lipid peroxidation and excitotoxicity. Interactions between astrocytes and microglia, macrophages, and endothelial cells promote neuroinflammation, blood–brain barrier disruption, synaptic and axonal dysfunction, and neuronal apoptosis via mediators such as matrix metalloproteinases, vascular endothelial growth factor, and adhesion molecules. Additionally, reactive astrocytes inhibit neural regeneration through glial scar formation and secretion of inhibitory molecules.

**Conclusions:**

By combining mechanistic studies with translational perspectives, this review highlights that astrocytes act as central mediators of secondary injury and repair in TBI pathology. Given the context‐dependent nature of astrocyte signaling, future therapeutic strategies should aim to reprogram astrocyte responses with temporal and cell‐type precision rather than pursuing broad inhibition. Also, targeting astrocyte‐specific pathways, such as the TLR4 and NF‐κB pathways, may mitigate secondary injury and improve outcomes. This underscores the therapeutic potential of modulating astrocyte responses in the treatment of TBI.

## Graphical abstract

TBI induces intrinsic astrocytic changes, including reactive astrogliosis, A1 and A2 polarization, ion channels activation, pathological edema, oxidative stress, and metabolic dysregulation. These injury‐responsive astrocytes regulate neuroinflammation and tissue remodeling through dynamic crosstalk with microglia, macrophages, vascular endothelial cells, neurons, oligodendrocytes, and neural stem cells. Multiple signaling pathways drive these astrocytic pathological changes following TBI.

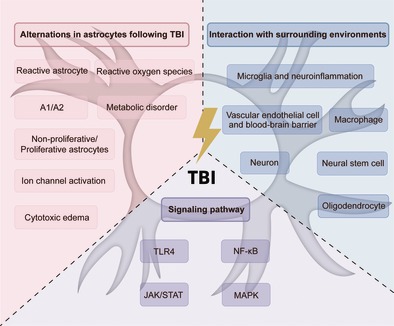

**Abbreviations**
11C‐DED: 11C‐deuterium‐l‐deprenyl
11C‐PIB: 11C‐Pittsburgh compound B
2‐AG: 2‐arachidonoylglycerol
ADAM‐10: a disintegrin and metalloproteinase domain‐containing protein 10
Aldh1l1: aldehyde dehydrogenase 1 family member L1
AMPA: α‐amino‐3‐hydroxy‐5‐methyl‐4‐isoxazolepropionic acid
AQP4: aquaporin‐4
ATF2: activating transcription factor 2
ATP: adenosine triphosphate
Aβ: amyloid‐beta
BDNF: brain‐derived neurotrophic factor
C1q: complement component 1q
C1ql1: complement C1q like 1
C3: complement component 3
C4b: complement component 4b
CaMK: calmodulin‐dependent protein kinase
CCL7: C‐C motif chemokine ligand 7
connexin 43: gap junction protein alpha 1
CREB: cAMP response element binding protein
CSF: colony‐stimulating factor
CSPGs: chondroitin sulfate proteoglycans
CXCL1: C‐X‐C motif chemokine ligand 1
CXCL10: C‐X‐C motif chemokine ligand 10
CXCR2: C‐X‐C motif chemokine receptor 2
DAMPs: damage‐associated molecular patterns
EAAT: excitatory amino acid transporter
EphA4: ephrin type‐A receptor 4
ephrinA3: ephrin A3
ERK: extracellular signal‐regulated kinase
ERK1/2: extracellular signal‐regulated kinase 1/2
ET‐1: endothelin‐1
ETA: endothelin A
ETB: endothelin B
Foxo3a: Forkhead box O3a
GDNF: glial cell line‐derived neurotrophic factor
GFAP: glial fibrillary acidic protein
GLAST: glutamate aspartate transporter
GLT‐1: glutamate transporter‐1
GluN1: glutamate ionotropic receptor NMDA type subunit 1
GluN2A‐D: glutamate ionotropic receptor NMDA type subunit 2A‐D
GluN3A‐B: glutamate ionotropic receptor NMDA type subunit 3A‐B
Gpc6: glypican 6
GTPases: guanosine triphosphatases
H_2_O_2_
: hydrogen peroxide
HMGB1: high‐mobility group box 1 protein
HSPs: heat‐shock proteins
ICAM‐1: intercellular adhesion molecule‐1
Ifi27: interferon alpha inducible protein 27
Ifit1: interferon induced protein with tetratricopeptide repeats 1
IFN: interferon
IGF‐1: insulin‐like growth factor 1
IKK: inhibitor of nuclear factor kappa‐B kinase
IL‐17: interleukin‐17
IL‐1β: interleukin‐1 beta
IL2R: interleukin‐2 receptor
iNOS: inducible nitric oxide synthase
IP3: inositol trisphosphate
Irf9: interferon regulatory factor 9
IκB: inhibitory protein kappa B
JAK: Janus kinase
JNK: c‐Jun N‐terminal kinase
JNKs: c‐Jun N‐terminal kinases
KLF11: Krueppel‐like factor 11
MAGL: monoacylglycerol lipase
MAP2K: mitogen‐activated protein kinase kinase
MAP3K: mitogen‐activated protein kinase kinase kinase
MAPK: mitogen‐activated protein kinase
Megf10: multiple EGF‐like domains 10
Mertk: MER proto‐oncogene, tyrosine kinase
MMPs: matrix metalloproteinases
MyD88: myeloid differentiation factor 88
NADPH: nicotinamide adenine dinucleotide phosphate
NF‐κB: nuclear factor kappa‐light‐chain‐enhancer of activated B cells
NGF: nerve growth factor
NgR1: Nogo receptor 1
NgR3: Nogo receptor 3
NKCC1: Na+‐K+‐2Cl− cotransporter 1
NLRP3: NOD‐like receptor family pyrin domain containing 3
NMDA: N‐methyl‐D‐aspartate
NO: nitric oxide
NOX: NADPH oxidase
Nrf2: nuclear factor erythroid 2‐related factor 2
Oasl2: 2′‐5′ oligoadenylate synthetase like 2
P2X7: purinergic receptor P2X7
P2Y1: purinergic receptor P2Y1
PAFR: platelet‐activating factor receptor
PAMPs: patterns associated with pathogens
PKC: protein kinase C
PLC: phospholipase C
PRRs: pattern recognition receptors
Ptgs2: prostaglandin‐endoperoxide synthase 2
PTPRS: protein tyrosine phosphatase receptor type S
PTX3: pentraxin 3
Rho‐GTP: Rho family GTPases
S100A10: S100 calcium binding protein A10
S100B: S100 calcium binding protein B
SAPKs: stress‐activated protein kinases
Smad2: mothers against decapentaplegic homolog 2
SND1: staphylococcal nuclease and Tudor domain containing 1
SPARC: secreted protein acidic and rich in cysteine
Sparcl1: SPARC like 1
STAT3: signal transducer and activator of transcription 3
TGF‐β: transforming growth factor beta
TLR: toll‐like receptor
TLR4: toll‐like receptor 4
TNF‐α: tumor necrosis factor‐alpha
TRAFs: TNF receptor‐associated factors
TREM‐2: triggering receptor expressed on myeloid cells 2
TrkB: tropomyosin receptor kinase B
TRP: transient receptor potential
TSPO: translocator protein
Tyk2: tyrosine kinase 2
TYMP: thymidine phosphorylase
VCAM‐1: vascular cell adhesion molecule‐1
VEGF‐A: vascular endothelial growth factor A
vWF: von Willebrand factor

## 1 Introduction

Astrocytes are the most abundant type of glial cells in the central nervous system (CNS), which establish a consistent network across the mammalian CNS [1]. Under normal physiological conditions, astrocytes typically have a distinctive star‐shaped cell body with numerous slender protrusions that extend to form connections with various cells as well as blood vessels [2]. In the cerebral gray matter, they envelop neuronal cell bodies and dendrites, while in the white matter, they align along nerve fibers. The protrusions terminate in swollen structures called endfeet, which attach closely to capillary walls and constitute a crucial component of the blood–brain barrier (BBB) [3]. Astrocytes also interact with oligodendrocytes, microglia, perivascular cells, meningeal fibroblasts, and circulating immune cells, to support the maintenance of normal brain function [4]. Moreover, they play pivotal roles in sustaining extracellular fluid, ion, and extracellular matrix homeostasis, supply glucose metabolites to neurons, regulate blood flow and interstitial fluid drainage, support mitochondrial integrity for energy maintenance, and modulate synaptic development [5, 6]. Thus, astrocytes have numerous vital roles in the stability of the CNS under normal physiological conditions.

Traumatic brain injury (TBI) is a significant and growing global health issue, being a leading cause of mortality and long‐term disability worldwide and affecting an estimated 69 million individuals annually [7]. It is caused by an external force, encompassing a highly heterogeneous spectrum of severity from mild concussion to severe, life‐threatening injuries. The pathophysiology of TBI is a dynamic process characterized by primary and secondary injuries. Primary injury refers to the immediate, irreversible mechanical damage resulting from the initial impact, including neuronal shearing, vascular disruption, and axonal tearing [8]. The instantaneous damage sets the stage for a more prolonged phase of damage. Secondary injury evolves over hours to months following the initial trauma, driven by excitotoxicity, oxidative stress, mitochondrial dysfunction, blood–brain barrier disruption, cerebral edema, and robust neuroinflammation [9]. Critically, secondary injury processes are estimated to account for up to 70% of the total neuronal damage, representing a critical therapeutic window for intervention [10].

Despite decades of research, the clinical management of TBI remains predominantly supportive, focusing on stabilizing vital signs and mitigating intracranial pressure. To date, no effective neuroprotective pharmacological therapy has been successfully translated to the clinic, underscoring the urgent need for novel therapeutic strategies [11].

Within this context, the role of glial cells, particularly astrocytes, has come into sharp focus. Following TBI, astrocytes undergo a dramatic transformation known as reactive astrogliosis. This response is double‐edged. Initially, reactive astrocytes attempt to seal the injury site, limit inflammation, and provide neurotrophic support. However, sustained and severe activation can lead to the formation of a glial scar, which, while isolating the damaged area, also creates a physical and chemical barrier to axonal regeneration [12]. Moreover, dysregulated astrocytes are key drivers of pathological processes such as cytotoxic edema and are major contributors to the persistent neuroinflammation by releasing pro‐inflammatory cytokines. Therefore, given their central role in orchestrating secondary injury cascades, astrocytes have emerged as pivotal and promising targets for developing innovative therapies aimed at modulating their responses to preserve neural function and promote repair after TBI.

This review will first elaborate on the phenotypic and functional changes of astrocytes post‐TBI, then analyze their activation mechanisms and intercellular interaction networks, and finally focus on core signaling pathways and therapeutic targets, providing a theoretical basis for TBI intervention.

## 2 Alterations in Astrocytes Following TBI

### 2.1 Reactive Astrocytes in TBI: Transcriptional Alterations, Reliable Markers, and Dual Roles

Following TBI, astrocytes exhibit various alterations in phenotype and function [3], resulting in beneficial as well as certain adverse effects. Astrocytes are categorized into different subtypes according to differences in gene expression, cellular morphology, and deleterious functions. For example, reactive astrocyte is a universal component of the neuroinflammatory response and a pathological hallmark of all CNS injuries [13, 14, 15]. Morphological and functional changes are ultimately due to changes in gene expression. Todd et al. identified persistent astrocyte‐specific transcriptional changes post‐TBI through RNA sequencing analysis of 19,122 genes. Enriched astrocyte populations exhibited upregulated levels of certain markers (e.g., Aldh1l1, AQP4, and GFAP). Witcher et al. similarly revealed TBI‐induced transcriptional alterations in astrocytes via single‐cell RNA sequencing. One week post‐injury, they identified 55 differentially expressed genes in astrocytes (50 upregulated; 5 downregulated); the upregulated genes included those encoding for indicators associated with astrocyte activation (e.g., GFAP, serpina3n, and vimentin), the type I interferon signaling pathway (e.g., Ifi27, Oasl2, Irf9, and Ifit1), and complement pathways (e.g., C4b and C1ql1) [16]. A separate investigation likewise noted increased Timp1 and Steap4 gene activity within reactive astrocytes [17].

Interestingly, changes in astrocyte gene expression following TBI have also been observed in non‐human species. Initial studies by Weber et al. demonstrated that *Xenopus laevis* tadpoles exhibit a reactive astrocyte response. They used quantitative RT‐PCR analysis of tadpole brain tissue samples to examine alterations in gene expression at different recovery time‐points following localized TBI and identified distinct temporal astrocyte‐related gene expression patterns. Specifically, Aldh1l1, GLAST, vimentin, and AQP4 expression showed transient upregulation peaking at 24 h post‐injury before returning to baseline levels within 7 days, whereas other genes exhibited delayed increases [18]. This indirectly suggests that TBI exerts a predisposing effect on gene expression in astrocytes and that the differential expression of astrocyte genes in tadpoles could provide us with research ideas for analyzing changes in human astrocytes after TBI.

Reactive astrocytes express many typical markers following TBI such as GFAP and TSPO [19, 20, 21, 22, 23, 24]. GFAP, a cytoskeletal protein specific to astrocytes, serves as a primary marker for astrocyte activation. GFAP levels effectively increase in activated astrocytes, which indicates that astrocyte activation and/or proliferation has occurred [25]. TSPO, significantly increasing in astrocytes after TBI, was identified as another marker. This was further validated by increased ligand binding of astrocyte‐targeted tracers such as 11C‐PIB and 11C‐DED [24].

Reactive astrocytes have a dual impact on the brain, manifesting as a continuum of progressive changes over time [21, 26]. Overactivation of astrocytes results in the production of bioactive cytokines that modulate pathophysiological responses, enhancing the inflammatory response, leading to glial scarring, and limiting neuronal regeneration and neuroplasticity. Nonetheless, increased production of neurotrophic factors supports neuronal survival, synapse formation, and neurogenesis in neural precursor cells [6]. This underscores the dual role of reactive astrocytes and could aid in the development of therapies aimed at improving tissue healing and reducing damage.

### 2.2 Multi‐Activation Mechanisms of Reactive Astrocytes Post‐TBI

Following TBI, astrocytes rapidly detect injury‐associated signals, including mechanical deformation, extracellular ionic fluctuations, and inflammatory mediators [27, 28, 29]. These signals activate distinct molecular pathways in astrocytes, driving reactive gliosis.

Mechanical injury constitutes the primary activation signal for astrocytes post‐TBI. Physical trauma disrupts astrocytic membrane integrity, triggering intracellular stress responses such as calcium influx. Rosa et al. employed dual fluorescence labeling with sulforhodamine 101 (a specific astrocyte marker) and Fluo4‐AM (a cell‐permeant calcium indicator). Fluorescence microscopy analysis revealed a significant calcium elevation (defined by sustained Ca^2+^ transients) in activated astrocytes within the ipsilateral cortical region post‐injury [30]. Elevated cytosolic Ca^2+^ levels activate calcium‐dependent signaling cascades (e.g., those involving calmodulin kinases) to initiate astrocytic activation processes [27, 28]. We will discuss this pathway further in Section 2.5, which describes ion channel‐mediated astrocyte activation in TBI.

Meanwhile, tissue damage releases pro‐inflammatory cytokines (TNF‐α, IL‐1β, IL‐17) from injured neurons and immune cells [29, 31], DAMPs released from damaged cells bind PRRs on astrocytes, particularly TLR4 [32], activating the TLR4 pathway and driving reactive astrogliosis [33, 34]. This leads to rapid astrocyte proliferation from the injury core to distal tissues [5]. TLR4 antagonists reduce post‐TBI astrocyte proliferation, confirming TLR4 pathway involvement [30].

**FIGURE 1:**
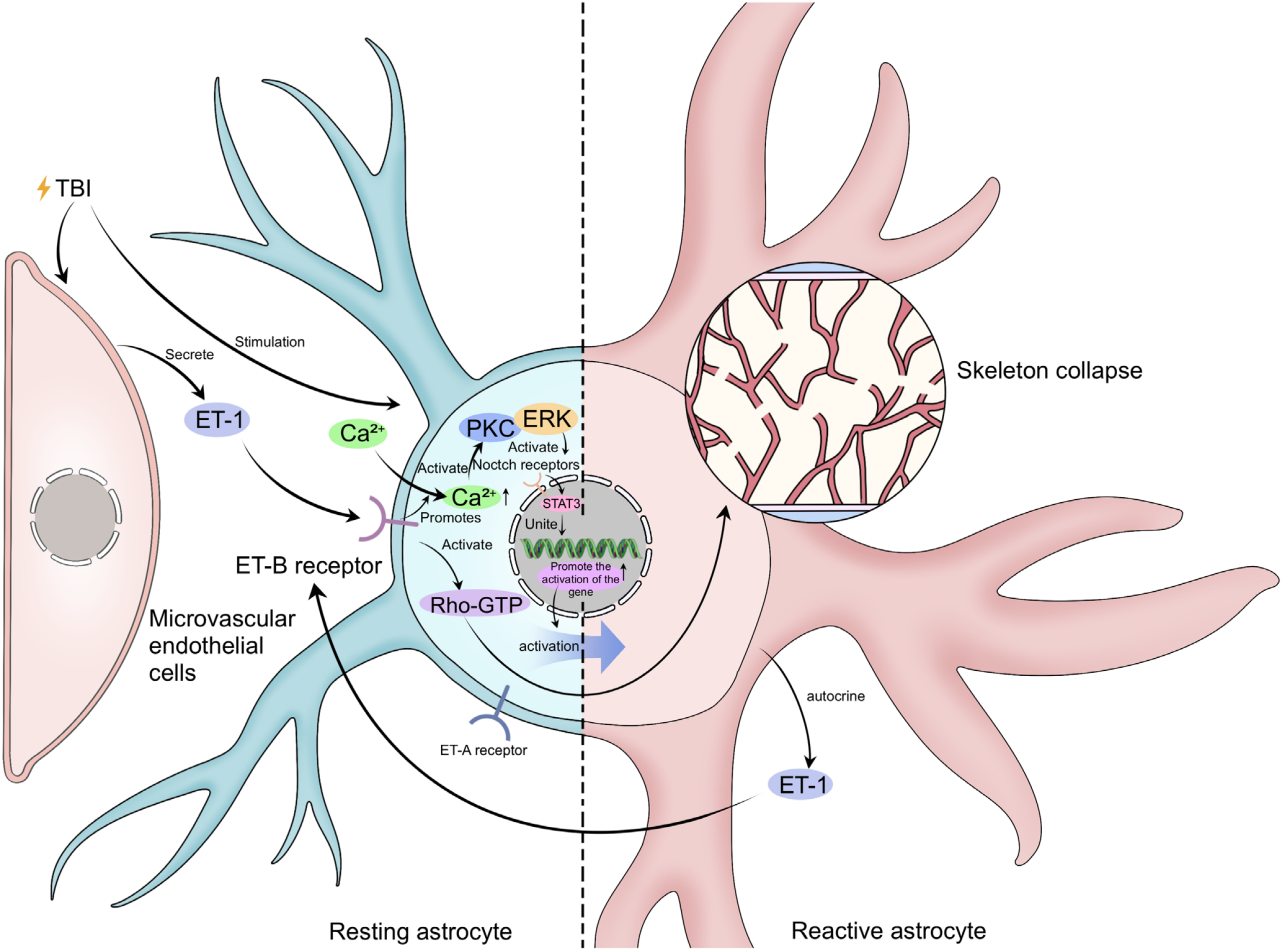
Mechanism of ET‐1/ET‐B receptor‐mediated PKC/ERK/Rho‐GTP signaling and STAT3‐driven reactive astrocytes activation in TBI. TBI stimulates microvascular endothelial cells to secrete ET‐1, which acts on the ET‐B receptor (which also involves the ET‐A receptor) in resting astrocytes. Activation of the ET‐B receptor leads to an increase in the level of intracellular Ca^2+^ and the subsequent activation of PKC and then ERK and Rho‐GTP. Together, these signaling transmissions lead to gene activation to drive the transformation of resting astrocytes to reactive astrocytes through pathways such as the STAT3 pathway, triggering cytoskeletal collapse. Furthermore, reactive astrocytes maintain or enhance their own activation state through an ET‐1‐mediated autocrine mechanism of propagating a continuous activation cycle.

The activation of astrocytes is also negatively involved in the Notch signaling and extracellular matrix remodeling, which is mediated by the ETB receptor [25, 26]. Astrocytes predominantly express ETB rather than ETA receptors. ETB activation elevates intracellular calcium, triggering PKC/ERK signaling, driving proliferation and secreting bioactive substances via STAT3, a key component of the JAK/STAT pathway (discussed in Section 4.3). ETB receptors also activate Rho‐GTP dependent cytoskeletal remodeling, facilitating phenotypic transformation into reactive astrocytes [35]. ETB receptor activation is mainly via ET‐1 [21, 35]. ET‐1 is synthesized by cerebral microvascular endothelial cells and reactive astrocytes, and its level is elevated in the injured brain. ETB receptor stimulation can upregulate precursor ET‐1 mRNA levels in astrocytes. This suggests that ET‐1 production by astrocytes is enhanced through an autocrine mechanism in brain diseases, further supporting that ETB receptors mediate activated astrocytes through the positive feedback circle [21]. These processes are illustrated in Figure 1. Interestingly, astrocytes activated via the ET‐1/ETB pathway continue to interact with vascular endothelial cells through this pathway, thereby triggering cerebral edema. This will be discussed in detail in Section 3.3.

Non‐classical pathways are also involved in astrocyte activation. Studies on PAFR−/− mice revealed a reduction in GFAP positive activated astrocytes in hippocamp post‐TBI, implicating the platelet‐activating factor's role in driving astrocyte activation and the associated inflammatory responses [36]. Furthermore, astrocytic phagocytosis of Aβ aggregates, characterized by GFAP‐positive cytoskeletal encapsulation, suggests Aβ may promote their activation [37]. Finally, blood can also induce astrocytic proliferation, GFAP expression, and reactive transition, potentially through albumin, thrombin, or fibronectin signaling [38, 39, 40].

Diverse injury‐related signals aforementioned converge on a limited set of core pathways in astrocytes. Inflammatory cytokines and DAMPs activate receptors such as TLR4, triggering NF‐κB, JAK/STAT3, and MAPK cascades (see Section 4 for details). These pathways act as central hubs that integrate extracellular stress signals into coordinated transcriptional programs, driving astrocytic responses. Understanding this integration is key to deciphering astrocyte heterogeneity and developing strategies to modulate astrocyte reactivity after TBI.

### 2.3 Functional Polarization of Reactive Astrocytes Post‐TBI: Neurotoxic A1 Versus Neuroprotective A2 Phenotypes

Post‐TBI, activated astrocytes exhibit either the A1 or A2 phenotype, which are defined by their opposing roles in neuroinflammation and neuroprotection. Both types play significant roles in scar tissue formation [41] and can be triggered by post‐injury microglia‐derived inflammatory signals [42, 43].

A2 astrocytes exhibit neuroprotective functions via upregulating neurotrophic factors (e.g., BDNF and NGF) and anti‐inflammatory mediators (e.g., IL‐10, IGF‐1, and TGF‐β) [44, 45]. Notably, the polarization toward the A2 phenotype is precisely regulated by specific cytokines such as IL‐4 and IL‐13, which are released by immune cells (e.g., M2 microglia, T cells) in the injured brain [46]. These cytokines signal drive the expression of characteristic A2 markers such as S100A10 and PTX3, through the IL‐4 receptor α (IL‐4Rα) and STAT6 pathway, thereby promoting a reparative astrocytic state [17]. They are capable of taking up and metabolizing glutamate to prevent excitotoxicity, thus regulating the extracellular ion balance to maintain normal neuronal electrical activity, and exerting anti‐inflammatory effects during neuroinflammation [47, 48]. A1 astrocytes, conversely, are driven by proinflammatory cytokines and C1q released from activated microglia, and play a crucial proinflammatory role in the post‐TBI inflammatory response [37, 49, 50, 51]. They express high levels of several inflammatory cytokine such as CXCL10, C1q, and C3, which in turn leads to the secretion of inflammatory mediators, including IL‐6, CXCL1, IL‐1β, IFN‐γ, and TNF‐α [45]. Notably, the expression of C3, which serves as a hallmark biomarker for A1 activation, is upregulated most significantly [52]. The overexpression of these complement components in A1 cells have toxic effects on surrounding neurons [53, 54]. For instance, high C3 expression in A1 astrocytes drives membrane attack complex (MAC) formation and disrupts neuronal integrity [55]. Pathologically, A1 reactive astrocytes lose critical homeostatic capacities, including synaptic support and phagocytosis, due to downregulation of phagocytic receptors (Mertk and Megf10) and synaptogenic factors (Gpc6 and Sparcl1) [50, 56].

While the A1/A2 dichotomy provides a valuable framework, it is important to acknowledge its limitations. Recent single‐cell RNA sequencing studies reveal that reactive astrocytes exhibit a spectrum of molecular signatures that extend beyond a strict binary classification [57], with some cells co‐expressing both A1 and A2 markers or displaying “intermediate” or “hybrid” phenotypes [47]. Furthermore, astrocyte polarization is highly dynamic and time‐dependent. The balance between A1 and A2 phenotypes can shift significantly across different phases of TBI, from the acute inflammatory phase (often A1‐dominant) to the subsequent repair phase (where A2 responses may become more prominent) [58]. This balance is also influenced by the specific TBI model used. For instance, the controlled cortical impact (CCI) model, which produces a focal injury, may elicit a different astrocytic response profile compared to the fluid percussion injury (FPI) model, which often involves more diffuse damage [59]. Variations in the A1/A2 ratio across these models highlight the context‐dependent nature of astrocyte reactivity and underscore the need for careful interpretation of findings from different experimental paradigms [60].

In conclusion, the A1/A2 dichotomy reflects astrocyte function duality in TBI—the A2 subtype promotes neuroprotection via trophic support and anti‐inflammatory activity, while the A1 subtype drives neurotoxicity through complement activation and proinflammatory signaling. However, the emerging complexity, including intermediate states and spatiotemporal dynamics, suggests a more nuanced reality. Astrocyte subtypes' interplay dictates TBI outcomes. Thus, targeting this balance may offer therapeutic strategies to curb neuroinflammation while preserving astrocytic repair potential.

### 2.4 Non‐Proliferative/Proliferative Astrocytes and Their Relationships With Scar‐Formation Post‐TBI

Reactive astrocytes can also be categorized as either proliferative boundary‐forming reactive astrocytes or non‐proliferative reactive astrocytes based on their migration pattern and involvement in scar formation [61]. Non‐proliferative reactive astrocytes are also called homomorphic astrocytes and are characterized by the presence of hypertrophied astrocytes that remain within a specific region. Alternatively, non‐homomorphic astrocytes, which are also called proliferative boundary‐forming astrocytes, proliferate and move toward a lesion and can create a glial scar by extending their protrusions [62]. After TBI onset, proliferative boundary‐forming astrocytes multiply by cell division for lesion boundary formation. These cells originate from localized astrocyte proliferation or from periventricular neural progenitor cells. However, non‐proliferative reactive astrocytes respond to injury through changes in morphology rather than proliferation, usually manifesting as cellular hypertrophy [63]. Crucially, the involvement of proliferative astrocytes is central to the structural consolidation of the glial scar, whereas non‐proliferative astrocytes may contribute through hypertrophy and molecular signaling without direct incorporation into the scar matrix [64].

Following TBI, astrocytes can rapidly sense injury‐related signals [65, 66, 67, 68]. The proliferative boundary‐forming reactive astrocytes sense these signals and increase cell body size as well as protrusion number and thickness. These protrusions extend toward the injury site, forming an initial network. At the same time, the astrocytes begin to proliferate to form glial scar, which is traditionally thought to be an obstacle to CNS repair [14]. Scar formation also requires the interaction of astrocytes with other cells [13, 69]. Stimulated microglia secrete pro‐inflammatory molecules and trophic agents, including TNF‐α, IL‐1β, and IL‐6 as well as IGF‐1, to stimulate the transition of astrocytes into hyperprolific, boundary‐forming reactive cell [70]. Furthermore, astrocytes and NG2 oligodendrocyte progenitors form rope‐like structures around the center of the inflammatory lesion [69] to drive scar tissue formation. Finally, all three cells function together, with NG2 oligodendrocytes forming the glial rim with astrocytes and microglia and without the need for differentiation [71].

While glial scarring serves a protective role during the acute phase of CNS injury, it becomes detrimental in the chronic phase by impeding axonal regeneration and neuronal network restoration because of permanent fibrotic tissue deposition [14, 61, 72, 73, 74, 75, 76]. Activated astrocytes drive scar maturation via three synergistic mechanisms: (1) secretion of extracellular matrix (ECM) components, including collagen (structural reinforcement), fibronectin (cell adhesion), and laminin (migration guidance), which collectively form a dense mechanical barrier [77]; (2) production of CSPGs that electrostatically stabilize scar architecture [13]; and (3) regulation by molecular mediators such as debrin (modulates β1 integrin trafficking for scar compaction) and GFAP (modulates cytoskeletal reorganization for lesion boundary formation) [13, 14, 73]. This process evolves through three temporally overlapping phases [13], each governed by distinct molecular events:

Initially (Phase 1: seconds to days post‐injury), astrocyte activation without morphological changes accompanies microglial/NG2 glia recruitment to injury cores. Key early molecular signals include extracellular ATP and glutamate, which trigger initial astrocytic Ca^2+^ signaling [78], alongside pro‐inflammatory cytokines released by microglia, priming astrocytes for subsequent proliferation and activation [79]. Subsequently (Phase 2: 2–10 days post‐injury), reactive astrocytes proliferate to encapsulate the lesion. This proliferative phase is critically dependent on the JAK/STAT3 signaling pathway (see Section 3.1 for details) that drives their division and migration [80]. Concurrently, molecules such as Notch signaling components further regulate the transition of astrocytes into a scar‐forming phenotype [81]. Ultimately (Phase 3: > 7 days post‐injury), ECM remodeling establishes permanent fibrotic scars characterized by GFAP^+^ astrocytic networks and CSPG‐rich matrices, completing the transition from adaptive containment to maladaptive regeneration blockade. During this phase, specific CSPG subtypes—such as aggrecan (ACAN) and versican (VCAN)—are prominently upregulated [74]. ACAN contributes to the structural stability and inhibitory nature of the scar, while VCAN influences cell adhesion and ECM assembly. Additionally, MMPs and their inhibitors (TIMPs) fine‐tune ECM composition and density, finalizing the scar's mature, inhibitory properties [82].

The formation of stable, long‐term scar tissue can result in a variety of detrimental effects on the brain. Thick scarring around axons has the same effect as insulation around electrical wires, impeding nerve impulse conduction. This interference can cause delays, interruptions, or errors in signal transmission [74]. Second, scar tissue compresses the blood vessels in the brain, reducing the internal diameter of the blood vessels and decreasing blood flow to eventually restrict the oxygen and glucose [83]. As a result, neurons receive inadequate nutritional support, impairing their normal metabolic functions.

### 2.5 Ion Channel‐Mediated Astrocytes Activation in TBI

Following TBI, astrocytes can be activated through multiple ion channels. The first mechanism is direct mechanical stimulation: the mechanical forces induced by TBI can directly damage astrocyte membranes, causing deformation of intracellular intermediate filaments. This deformation activates mechanosensitive ion channels and receptors such as TRP ion channels and mechanosensitive potassium channels [84]. Mechanical shear stress alone has been reported to induce activation of mechanosensitive calcium channels [85]. Activation of these channels results in the influx of sodium and calcium into astrocytes, which in turn leads to ATP release through calcium‐dependent and calcium‐independent manners [86, 87, 88, 89]. This mechanical stimulation also has the potential to affect the connectivity of astrocytes with surrounding cells, particularly through gap junctions. Gap junctions are composed primarily of connexin proteins, enabling the exchange of ions and small molecules between adjacent cells. The mechanical deformation may alter their permeability and consequently have an impact on the flow of ions within the astrocyte network [90, 91, 92].

Damaged neurons also release excessive glutamate and ATP, activating astrocytic receptors and ion channels. To counteract excitotoxicity, astrocytes undergo morphological remodeling, developing branched processes that envelop synapses. These specialized structures upregulate the potassium channel Kir4.1 and GLT‐1/EAAT, enabling efficient extracellular potassium and glutamate clearance [93, 94, 95]. Glutamate uptake through EAATs [96] drives Na^+^ influx and K^+^ efflux, altering intracellular ion balance and membrane potential, while receptor‐mediated depolarization further activates voltage‐gated ion channels [97, 98]. Notably, ion and neurotransmitter homeostasis are tightly coupled: rapid K^+^ buffering sustains the electrochemical gradient required for glutamate transport, whereas glutamate uptake depends on the Na^+^ gradient maintained by Na^+^/K^+^‐ATPase activity [99]. Following TBI, the downregulation of Kir4.1 combined with reduced GLT‐1 expression (see Section 2.8.2) collectively disrupts the “ion‐glutamate” homeostasis, constituting a key mechanism of excitotoxic injury.

Concurrently, ATP binds to astrocytic purinergic receptors (e.g., ionotropic P2Y1), inducing calcium influx and subsequent activation of calcium‐dependent enzymes like Ca^2+^/CaMK, NLRP3 inflammasomes and certain phosphatases. These enzymes modulate inflammatory gene expression and promote cytokine/chemokine production (see Section 3.1) [52, 87, 100, 101]. Calcium signaling could also propagate through dual context‐dependent mechanisms: (1) physiological synchronization occurs via Cx43 gap junctions, allowing rapid (< 100 ms) IP3 transfer and localized calcium release (≤ 100 μm) [102]; (2) pathological conditions (e.g., ischemia) favor ATP‐dependent paracrine signaling, where pannexin‐1‐mediated ATP release activates neighboring P2Y1/P2X7 receptors, triggering PLC‐IP3 cascades for long‐range propagation (> 200 μm) and glial hyperactivation [103]. This transition involves Cx43 phosphorylation‐induced gap junction closure, establishing ATP dominance in disease states [104]. The interplay of these spatiotemporally regulated mechanisms drives neuroinflammatory and excitotoxic cascades following TBI.

### 2.6 Astrocytes‐Mediated Pathological Edema in TBI

Cytotoxic edema, which is characterized by the swelling of cells, especially astrocytes, due to water retention, is a prominent feature of the damaged brain after TBI. Excessive water retention increases the water content of the brain, which in turn increases intracranial pressure (ICP). ICP beyond the normal range (70–200 mmH_2_O) can compress cerebral vasculature and reduce blood flow, which compromises oxygen/glucose delivery, disrupting neuronal metabolism and function [105]. Cytotoxic edema also disrupts cerebrovascular autoregulation—the dynamic adjustment of vessel diameter to ensure stable perfusion—which induces regional ischemia‐hypoxia, exacerbating secondary neuronal injury [106, 107]. Together, these damaging effects highlight astrocytes as critical therapeutic targets for mitigating TBI‐induced edema and its neuropathological consequences.

Astrocyte‐mediated ionic homeostasis plays a critical role in cytotoxic edema, and TBI has been shown to disrupt this homeostasis through multiple pathways. As mentioned in Section 2.5, downregulation of the inwardly rectifying potassium channel Kir4.1 leads to extracellular K^+^ accumulation and the establishment of an osmotic gradient, which drives water influx into the extracellular space and exacerbates vasogenic edema [40]. Meanwhile, cytotoxic edema arises from ion transporter dysregulation—upregulation of Na^+^‐K^+^‐2Cl^−^ cotransporter 1 (NKCC1) in astrocytes promotes intracellular Na^+^/Cl^−^ accumulation, inducing osmotic swelling [25, 108, 109]. siRNA‐mediated NKCC1 silencing can attenuate this swelling, confirming its pathological role [110].

AQP4, a water channel expressed mainly in astrocytes, is strongly linked to cytotoxic edema. Zhang et al. reported that FPI enhanced AQP4 expression, and that this increase induced astrocyte swelling in vitro [111]. Moreover, nuclear translocation of Foxo3a in astrocytes upregulates AQP4 expression, and Foxo3a knockout reduced cytotoxic swelling via AQP4 induction inhibition [112], suggesting that the Foxo3a‐AQP4 axis mediates cerebral edema. Kitchen et al. further demonstrated the expression and subcellular localization of AQP4 at the blood‐spinal cord barrier (BSCB). They also discovered that calmodulin bound directly to the carboxyl terminus of AQP4 and prompted its localization on the cell surface in a rat spinal cord injury model, which in turn aggravated TBI‐induced edema [113].

Furthermore, TBI activates the NF‐κB pathway, which transcriptionally upregulates both NKCC1 and AQP4, synergistically amplifying astrocyte swelling and edema progression [25, 108, 109, 110, 114]. The above ion/water channel disorders mediated by astrocytes share a common upstream transcriptional regulatory hub, NF‐κB. After TBI, the activated NF‐κB p65 subunit translocates into the nucleus and can directly bind to the κB sites in the promoter regions of the NKCC1 and AQP4 genes to initiate their transcription (see Section 4.2 for details). This mechanism directly couples neuroinflammatory signals with ion/water homeostasis imbalance, explaining why anti‐inflammatory therapy can simultaneously alleviate cytotoxic edema—essentially, it blocks the transcriptional activation of downstream effector molecules by NF‐κB.

Beyond the ion channels, others are also critically involved in brain edema caused by astrocytes after TBI. The basal membranes of astrocytes (such as laminin and collagen) are interconnected with cerebrovascular endothelial cells, which are a key component of the BBB. Mechanical forces can directly impair the basal membrane and lead to cerebral blood vessel rupture, resulting in the leakage of blood components (including proteins and fluids) outside the vessels. This blood components can in turn damage the basal membrane of astrocytes to further disrupt BBB integrity, leading to increased permeability. The increased degree of BBB leakage is closely related to brain edema [115, 116]. Downregulation of expression or mislocalization of tight junction proteins (e.g., claudin‐5 and occludin) can contribute to the leakage of plasma components into the brain parenchyma, thereby triggering cerebral edema of vasogenic origin [117].

In conclusion, brain edema is a complex pathological response triggered by TBI, and astrocytes are central in its development. Alterations in ion transporter protein activity, changes in BBB integrity, and abnormalities in water channel regulation contribute to the development of vasogenic and cytotoxic edema, which highlights several potential therapeutic targets for mitigating secondary brain injury.

### 2.7 Dysregulation of Astrocytic ROS Homeostasis in TBI

Astrocyte‐mediated mitochondrial reactive oxygen species (ROS) level regulation is critical for maintaining neuronal redox homeostasis, supporting metabolic stability and physiological nervous system functions in the CNS [118, 119]. However, TBI can disrupt astrocyte redox homeostasis through ROS overproduction and disruption of antioxidant defense systems.

ROS generation primarily stems from mitochondrial dysfunction and inflammatory activation. Mitochondria are essential for energy synthesis but can also act as sources of pathogenic ROS due to electron escape from compromised respiratory chain complexes I and III. These electrons react with oxygen to form superoxide anions (O_2_^−^), which propagate further ROS generation [120, 121]. At the same time, post‐TBI inflammation results in the release of cytokines (e.g., IL‐1β, TNF‐α) that activate NOX in astrocytes. NOX transfers electrons from NADPH to oxygen, producing O_2_^−^ and H_2_O_2_, thereby amplifying ROS levels and establishing a ROS‐inflammatory feedback loop that further exacerbates injury [122]. Besides, TBI‐mediated blood vessel rupture can result in an influx of fibrinogen into the brain to activate astrocytes, resulting in increased expression of TrkB. TrkB stimulation initiates NO production and subsequent nitrotyrosine deposition. NO can also increase intracellular ROS levels, ultimately causing further neuronal damage [38, 123, 124, 125, 126].

In terms of scavenging mechanisms, under normal conditions, astrocytes possess an antioxidant defense system consisting of superoxide dismutase (SOD), glutathione peroxidase (GSH‐Px), and catalase (CAT) [127, 128]. However, the activities of these antioxidant enzymes are negatively affected after TBI. For example, SOD converts O_2_^−^ into H_2_O_2_, but post‐TBI its activity is reduced due to the denaturation and loss of metal ion cofactors, rendering it unable to scavenge excessive O_2_^−^ in a timely manner [129, 130]. Additionally, GSH synthesis is suppressed via inhibition of γ‐glutamylcysteine synthetase, while ROS overconsumption depletes GSH reserves, crippling antioxidant capacity [128, 131]. The combined failure of ROS generation control and scavenging systems establishes a self‐amplifying cycle of astrocyte dysfunction and neuronal death.

**FIGURE 2:**
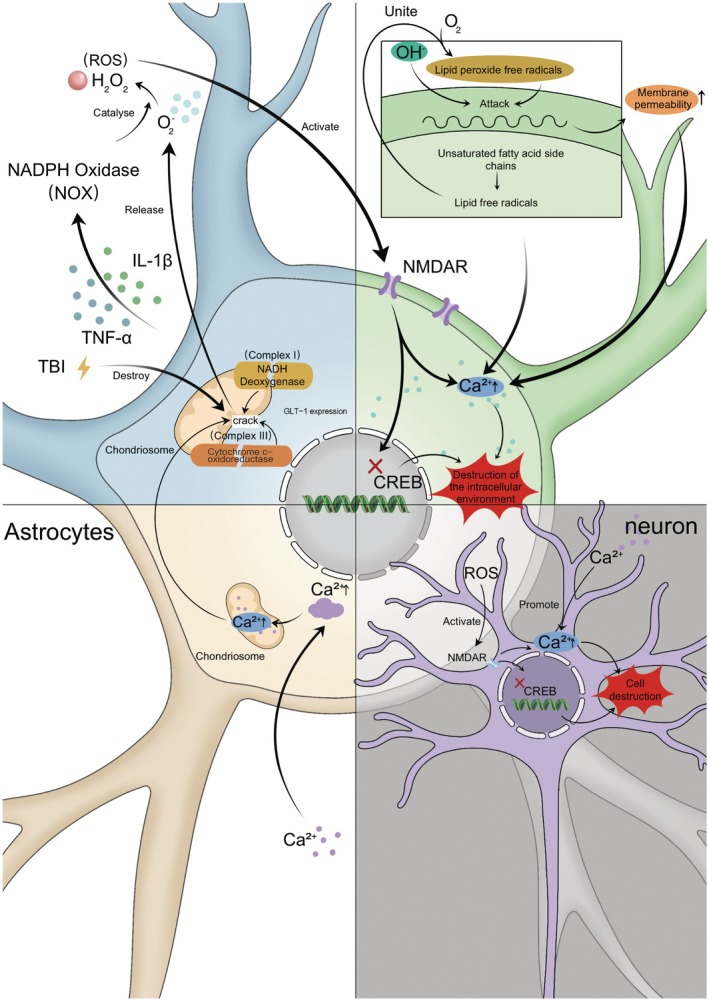
TBI‐induced astrocytic ROS cycle and ROS/mitochondria‐driven neuronal injury. TBI disrupts astrocytic mitochondrial respiratory chain complexes I and III, triggering electron leakage and their reaction with oxygen to generate O_2_^−^, which promotes the generation of ROS. At the same time, inflammatory factors (IL‐1β, TNF‐α) activate NOX, which also catalyzes the generation of ROS such as H_2_O_2_. Thereafter, ROS activate the NMDA receptor, which triggers an increase in Ca^2+^ influx, inhibits CREB, and disrupts intracellular homeostasis, leading to neuronal death. Additionally, hydroxyl radicals attack astrocyte membrane lipids, triggering a chain reaction of lipid peroxidation, increased membrane permeability, disrupted ionic homeostasis (including Ca^2+^ influx), and exudation or infiltration of cellular constituents in an abnormal manner, all of which exacerbate cellular damage and can lead to further mitochondrial dysfunction. Ultimately, this results in a vicious cycle of ROS overproduction.

ROS can induce multiple types of brain damage. Birnbaum et al. have shown that elevated levels of ROS are a key factor in the early progression of CNS disorders [132]. As shown in Figure 2, excessive ROS‐mediated damage arises from unregulated oxidative stress initially, including lipid peroxidation, which abstracts hydrogen atoms from unsaturated fatty acids in phospholipid bilayers. This generates lipid radicals that react with oxygen, perpetuating a chain reaction that reduces membrane fluidity and increases permeability. The resulting membrane compromise leads to pathological disruptions in calcium influx and facilitates leakage of intracellular components (enzymes, neurotransmitters, etc.) while permitting extracellular toxin entry, exacerbating cellular damage [133]. This can also lead to protein carbonylation and DNA strand breaks, which promote neuroinflammation, linking acute oxidative damage to chronic neurodegeneration [134]. Subsequently, during the acute phase of TBI, NOX2 activation propagates ROS‐mediated excitotoxicity to adjacent neurons and astrocytes [135], while chronic astrogliosis sustains ROS production, inducing persistent neuronal injury. Finally, elevated levels of ROS can activate NMDA receptors, amplifying oxidative stress in the CNS post‐TBI [131]. Activation of NMDA receptor activity leads to an increase in intracellular calcium influx and then inhibits CREB, ultimately contributing to neuronal cell death [136, 137]. It also results in mitochondrial dysfunction, driving further ROS overproduction and establishing a self‐amplifying cycle.

In short, TBI‐induced ROS dysregulation involves a complex interplay of mitochondrial impairment, inflammatory activation, and antioxidant defense failure. ROS‐mediated damage through lipid peroxidation, excitotoxicity, and NMDA receptor activation highlights the centrality of oxidative stress in both acute and chronic TBI. Strategically addressing these pathways could provide therapeutic avenues to reduce secondary neuronal damage.

### 2.8 Metabolic Disorders of Astrocytes After TBI

In physiological states, astrocytes play a supportive role in neural metabolism through their extensive network of gap junctions. These intercellular connections facilitate the propagation of metabolic waves through the astrocyte network, thus enabling remote metabolic coordination [138]. Furthermore, gap junctions facilitate the direct exchange of ions, nutrients, and signaling molecules, thereby contributing to the delivery of nutrients to neurons, the buffering of potassium and glutamate, calcium wave spreading, and the transmission of death signals within this network [139]. However, in post‐TBI conditions, astrocytes interfere with brain metabolic processes, which in turn causes multiple types of damage to the brain. The mechanisms involved are complex and extensive, affecting several key aspects of the brain, ultimately leading to neuronal and overall brain dysfunction [106, 140]. For example, TBI‐induced mitochondrial dysfunction and ROS overproduction mentioned above significantly impair astrocytic metabolic functions. The damage to mitochondrial integrity and electron transport chain components not only exacerbates oxidative stress but also disrupts ATP synthesis and metabolic coupling between astrocytes and neurons [141]. Experimental evidence also suggests that mitigating mitochondrial ROS can improve cellular metabolism [142]. This disruption is particularly evident in the dysregulation of lactate shuttling and glutamate metabolism, as detailed in the following subsections.

#### 2.8.1 Disturbed Energy Metabolism of Astrocytes Post‐TBI

In the resting state, astrocytes contribute to roughly 30% of the oxidative metabolism in the brain and about 50% of the glycolysis, with glycogenolysis occurring at a low, steady rate [139, 143]. This metabolic distribution underscores the distinctive and interrelated functions of astrocytes and neurons. Any disruption of these processes due to trauma can rapidly impair neuronal function, which may explain the pathophysiological mechanisms of gliosis and neurodegeneration after brain injury [144].

Lactate metabolism significantly contributes to brain injury after TBI [145]. The astrocyte‐neuron lactate shuttle (ANLS) model proposed by Magistretti and Pellerin states that lactate is the primary energy substrate for neurons during aerobic activation [146], although this model remains debated. Based on enzyme kinetic analysis results, Chih et al. argued that neurons primarily rely on extracellular glucose during heightened activity, with lactate acting as a supplementary fuel source during recovery phases and under pathological conditions such as TBI, where astrocyte metabolism is impaired [147]. Conversely, Jolivet et al. employed systems biology to reveal astrocyte–neuron metabolic interdependence—specifically, they proposed that astrocytes exhibit glucose uptake exceeding oxidative needs, converting excess glucose to lactate via glycolysis, which is exported for neuronal oxidation during activation in a process aligning with ANLS [148]. Barros and Deitmer further detailed astrocytic glycolysis and lactate transport as critical for intercellular metabolic coupling [139]. Collectively, ANLS operates in a context‐dependent manner, predominantly under high‐energy demand and pathological conditions rather than in the baseline physiological state. Following TBI, astrocytes maintain normal glucose oxidation and lactate release, but disrupted astrocyte‐neuron communication impairs neuronal lactate uptake, leading to extracellular lactate accumulation [149]. Elevated extracellular lactate levels disrupt pH homeostasis, exerting neurotoxic effects, while therapeutic strategies targeting lactate clearance show neuroprotective potential [150]. Thus, ANLS functionality appears contingent on intact astrocyte‐neuronal coordination, with its dysregulation in TBI contributing to metabolic crisis and neuronal injury.

#### 2.8.2 Astrocytes‐Mediated Disruption of Glutamate Metabolism Post‐TBI

Glutamate is the main excitatory neurotransmitter within the CNS and plays a vital role in synaptic plasticity and neuronal communication. However, excess glutamate in the extracellular space can cause neurotoxicity, which in turn can trigger excitotoxicity to result in extensive neuronal damage [151, 152, 153, 154]. Research also indicates a reduction in transporter protein efficacy following TBI. Activation of signaling pathways, such as the Akt and PKC pathways, negatively regulates the expression of the astrocyte glutamate transporters EAAT1 (GLT‐1 in humans) and EAAT2 (GLAST in humans), which impairs glutamate reuptake by astrocytes. Besides, IL‐1 is regarded as a pathogenic mediator, and it not only increases synaptic glutamate release but also impairs the uptake of extracellular glutamate by astrocytes via a calcium/PKC‐mediated mechanism. IL‐1 can also promote the activity of cystine/glutamate reverse transporter protein (system Xc–), which exacerbates the release of glutamate from astrocytes, thereby exacerbating excitotoxicity [155].

The resulting accumulation of glutamate overactivates glutamate receptors, particularly the NMDA receptor, which are key mediators of excitotoxic injury. The NMDA receptor consists of heterotetrameric subunits (GluN1, GluN2A‐D, and GluN3A‐B), which are responsible for regulating ionic flux, including calcium, sodium, and potassium ion flux. Furthermore, its subunit composition affects receptor function—GluN2B‐containing receptors are associated with the propagation of excitotoxicity, whereas GluN2A‐containing receptors have neuroprotective properties. Post‐TBI, there is an increase in the concentrations of glutamate that hyperactivate synaptic and extrasynaptic NMDA receptors, as well as other glutamate receptors such as the AMPA receptor [156, 157]. Overactivation of these receptors increases calcium inward flow [158], which activates cystatin and calpain‐dependent apoptotic pathways and further drives neuronal apoptosis and DNA damage.

Additionally, astrocytes regulate synaptic glutamate levels through EphA4 signaling. EphrinA3 deficiency is associated with an increase in astrocyte glutamate transporter activity, whereas EphA4 deficiency decreases synaptic glutamate availability. Dysregulation of this signaling axis impairs long‐term potentiation (LTP) and excitatory synaptic transmission, which in turn leads to neuronal dysfunction after TBI [159].

**FIGURE 3:**
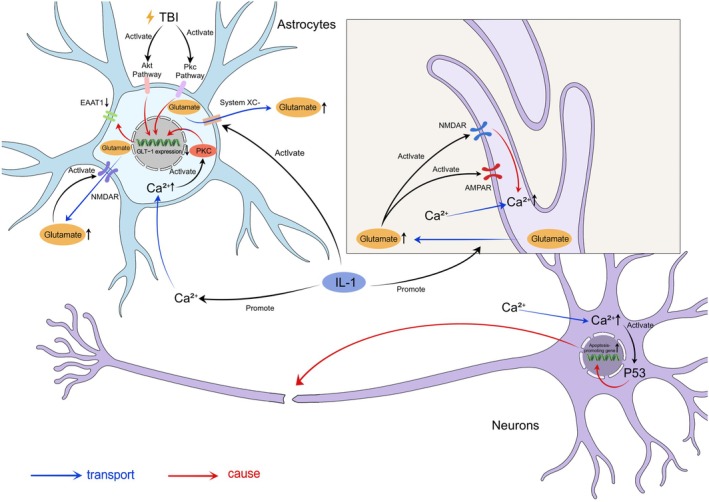
TBI‐driven Akt/PKC‐GLT‐1 dysregulation and glutamate excitotoxicity in neuronal apoptosis. TBI activates the Akt and PKC pathways in astrocytes and negatively regulates GLT‐1 expression, resulting in a decrease in glutamate reuptake by EAAT1 (corresponding to GLT‐1) and an accumulation of extracellular glutamate. At the same time, IL‐1 impairs the uptake of glutamate in astrocytes through a calcium/PKC‐mediated mechanism and promotes system XC‐activity, which exacerbates glutamate release from astrocytes. Glutamate release is also triggered by overactivation of NMDA receptors on astrocytes. At the neuronal level, extracellular accumulation of glutamate overactivates NMDA receptors and AMPA receptors at synapses, triggering a massive inward flow of Ca^2+^, activation of calcium‐dependent p53, and expression of neuronal apoptosis‐related genes, leading to apoptosis, structural damage, and disruption of neural circuits, ultimately resulting in CNS dysfunction.

Together, these processes result in extensive neuronal death, disruption of neural circuits, and long‐term CNS dysfunction [13, 160]. Most importantly, the overactivation of NMDA receptors on astrocytes triggers glutamate release, allowing excitotoxicity to persist in a vicious cycle [161, 162, 163] (Figure 3).

In conclusion, astrocytes are pivotal in modulating brain metabolism and glutamate balance via intricate and widely connected pathways. Post‐TBI, these homeostatic processes are severely disrupted, which in turn triggers neuronal energy defects, excitotoxicity damage, and widespread neurodegeneration. Mechanisms such as impaired lactate shuttling, downregulation of glutamate transporters, and exacerbated receptor overactivation highlight the impact of astrocyte dysfunction on the pathophysiology of TBI. A comprehensive analysis of these mechanisms can guide the formulation of specific therapeutic interventions to protect astrocyte function, correct metabolic disturbances, attenuate neuronal damage, and ultimately improve the prognosis of patients with TBI.

## 3 Altered Astrocytic Interaction With the Surrounding Environment After TBI

### 3.1 Astrocyte‐Microglia Interactions and Neuroinflammation Post‐TBI

Astrocytes and microglia are the two primary types of glial cells in the CNS [164, 165, 166, 167]. Microglia are involved in the major defense mechanisms of the brain [168, 169]. Post‐TBI, they are able to rapidly sense DAMP release from damaged neurons. Subsequently, they transition from a quiescent state to an activated state, which is accompanied by a change in their morphology from a long, branched quiescent form to a short, thick, amoeboid activated form [170, 171, 172, 173]. The mechanisms of interactions between microglia and astrocytes after TBI have been explored extensively. Witcher et al. detected an increase in rod microglia (Iba1‐positive) and astrocyte reactivity (detected based on GFAP) after TBI [172], which suggests a correlation between microglia and astrocytes. This section focuses on astrocyte‐microglia interactions and consequent neuroinflammation.

Astrocytes regulate microglial activity bidirectionally through cytokine‐mediated crosstalk—specifically, by secreting pro‐inflammatory factors to amplify microglial activation [174, 175]. Post‐TBI, astrocytes increase IL‐33 production, promoting microglial aggregation at injury sites [22], and secrete CCL7 to drive pro‐inflammatory mediator release from microglia [176]. Astrocytes also enhance microglial neurotoxicity, including Nurr1‐mediated dopamine toxicity, through NF‐κB‐dependent mechanisms [177]. Conversely microglia‐derived TNF‐α/IL‐1α reciprocally induce astrocyte reactivity via NF‐κB and JAK‐signal transducer and activator of transcription (e.g., STAT) signaling, upregulating inflammatory genes (e.g., C3), ROS and reactive nitrogen species levels in astrocytes [52, 173, 178], further exacerbating neuroinflammation.

This mutual reinforcement established a damaging positive feedback loop, generating a neurotoxic network marked by amplified inflammation, oxidative stress, and neuronal apoptosis/necrosis, and ultimately resulting in the disruption of neural circuits and accelerated neurodegeneration [179, 180]. Nonetheless, the interaction dynamics are context‐dependent; for example, Cdc42 depletion in TBI models was shown to reduce astrocyte recruitment, triggering compensatory microglial hyperactivity, whereas ischemic conditions induced excessive astrocyte activation that suppressed microglial responses via impaired anti‐inflammatory signaling [93, 177, 181, 182].

Neuroinflammation following TBI is also a major mechanism leading to brain damage. It is an extremely complex process in which astrocytes play a key mediating role, and complex communications between cells and molecules have a profound impact on prognosis after TBI [155]. Numerous pathways play crucial mediating roles in neuroinflammation, among which the TLR4 pathway is the most significant. As shown before, TBI resulting in the release of DAMPs activates PRRs such as TLRs for advanced glycation end products (RAGE) on astrocytes, which are central to initiating neuroinflammatory responses [10, 14, 15, 22, 51, 104, 162, 183, 184]. DAMP‐PRR interactions initiate the TLR4 signaling axis and then trigger certain intracellular signaling cascades, notably the NF‐κB and MAPK pathways (see Sections 4.1 and 4.2 for details), to drive the production of pro‐inflammatory cytokines (e.g., IL‐1β, IL‐6, TNF‐α), chemokines, and immune receptors [20, 22, 169, 184, 185].

Concurrently, ATP released from damaged cells establishes a chemotactic gradient, directing immune cells to injury sites and intensifying localized inflammation [186, 187]. Besides, as mentioned above, extracellular ATP can bind to purinergic receptors on astrocytes and microglia, inducing rapid elevation of intracellular Ca^2+^ levels [188, 189, 190, 191], activating NLRP3 inflammasomes—which are multiprotein complexes critical for sensing danger signals such as Ca^2+^ flux, ion efflux, ATP, and ROS [10, 13]—and promoting NLRP3 oligomerization, ASC recruitment, and caspase‐1 activation, thereby resulting in the cleavage of pro‐IL‐1β and pro‐IL‐18 into their active forms to amplify inflammation [10]. Additionally, extracellular Ca^2+^ influx results in early Ca^2+^ dynamics, further modulates astrocyte function, activates inflammatory signaling pathways, and enhances cytokine release [13, 191, 192]. Collectively, these mechanisms highlight the interplay between DAMP signaling, astrocyte activation, and Ca^2+^‐dependent pathways in driving post‐TBI neuroinflammation.

The complement system, another critical contributor to neuroinflammation following TBI, also interacts closely with astrocytes. Some studies have revealed elevated levels of complement components (such as C1q, C3, fB, and MACs) after TBI onset [169]. The activation of the classical, alternative, and lectin pathways results in the generation of various bioactive components, including C3a, C5a, and C3b [193]. Next, C3a and C5a bind to their respective receptors (C3aR and C5aR) on astrocytes, triggering intracellular signaling cascades that enhance astrocyte activation and drive the release of inflammatory mediators. In contrast, C3b facilitates phagocytic clearance of pathogens and damaged cells [194]. As for the MACs, which are formed during complement activation, they disrupt cell membrane integrity to induce cellular damage or death. Persistent complement activation in the chronic post‐TBI phase sustains astrocyte and microglial activation, fostering a chronic inflammatory state that impairs neuronal survival and functional recovery [14, 195, 196, 197]. This highlights the dual role of the complement system in both acute neuroinflammatory responses and chronic neurodegeneration following TBI.

Studies have also found elevated levels of circMETTL9, a circular RNA, in TBI models, notably within astrocytes. CircMETTL9 binds to and enhances the expression of SND1 and upregulates inflammatory molecules such as CXCL10 and CCL3, thereby enhancing the neuroinflammatory response. Moreover, knockdown of circMETTL9 significantly attenuated TBI‐induced neurological deficits and neuronal apoptosis, suggesting that it is a key initiator of astrocyte‐mediated neuroinflammation [198].

Furthermore, MAGL inhibition could also prevent the degradation of the 2‐AG to reduce NF‐κB activity and attenuate neuroinflammation [199]. From a therapeutic perspective, inhibition of thrombin or MAGL activity, as well as blockade of intercellular adhesion molecule‐1 (ICAM‐1), has shown promise in reducing neuroinflammation [200].

In conclusion, astrocytes mediate an extremely complex neuroinflammatory response after TBI through interactions with microglia as well as multiple astrocyte‐specific mechanisms, and enhanced insight into these mechanisms is expected to facilitate the development of innovative treatment strategies.

### 3.2 Astrocyte‐Macrophage Interactions and Neuroinflammation Post‐TBI

Following TBI, DAMPs released from injured neurons recruit macrophages to the lesion site, where they synergize with astrocytes to amplify neuroinflammation. Two distinct macrophage populations contribute to this process: brain‐resident macrophages and peripherally derived infiltrating macrophages [201, 202, 203, 204]. While DAMPs activate astrocytes into a reactive state, their other pathological role lies in facilitating macrophage‐astrocyte crosstalk, which drives sustained cytokine release and inflammatory cascades, ultimately exacerbating secondary neural damage.

In TBI, astrocytes become activated and increase IL‐33 production, promoting macrophage accumulation at the site of injury [171]. Activated astrocytes also release inflammatory mediators, including IL‐6 and TNF‐α, which enhance macrophage activation [205], promoting a more pronounced inflammatory state. The activated macrophages in turn release inflammatory factors such as IL‐1β, establishing an amplified inflammatory cycle with astrocytes [206]. Although this process helps to remove necrotic tissue and pathogens from the site of injury, excessive inflammation can damage surrounding healthy tissue.

In addition, astrocytes exhibit increased TREM‐2 expression after brain injury, which causes macrophages to switch to a more pro‐inflammatory phenotype [104]. Saber et al. found that global Trem2 knockout mice exhibited diminished brain macrophage activation, but astrocyte proliferation remained unaffected, leading to improved preservation of the hippocampus and improved behavioral outcomes in the long term after TBI [207]. These findings suggest that upregulation of astrocyte TREM‐2 enhances macrophage activation and may lead to excessive immune responses.

### 3.3 Astrocyte‐Vascular Endothelial Cell Interactions and BBB Damage After TBI

The BBB maintains cerebral homeostasis by selectively regulating the molecular exchange between blood and brain tissues, excluding neurotoxic substances, drugs, peptides, and peripheral components to help preserve neural function [208, 209]. Vascular endothelial cells are at the center of BBB integrity and are primary targets in TBI, where mechanical disruption and inflammatory cascades compromise tight junctions and transporter systems, followed by tissue damage, edema, inflammation, and neurological deficits [210]. Astrocyte‐endothelial interactions are critical for BBB maintenance [211, 212]. Post‐TBI, activated astrocytes and dysregulated crosstalk (e.g., aberrant cytokine release and loss of astrocytic endfeet coverage) exacerbates barrier permeability [40]. Deciphering these interaction mechanisms is pivotal for developing therapies to mitigate TBI‐induced BBB damage and secondary neuronal injury.

Specifically, astrocytes release inflammatory cytokines, including IL‐1β and TNF‐β, which initiate neuroinflammatory cascades [213, 214]. Notably, Aβ also induces a sustained elevation of astrocytic IL‐1β levels over time, promoting the secretion of IL‐1α, IL‐6, and TNF‐α from microvascular endothelial cells, thus exacerbating neuroinflammatory and endothelial responses [215, 216]. Concurrently, astrocyte‐derived IL‐1β upregulates adhesion molecules on cerebrovascular endothelial cells, such as ICAM‐1 and VCAM‐1, which selectively bind to integrins on monocytes and lymphocytes [217]. This interaction facilitates leukocyte adhesion to the inflamed endothelium, amplifying immune cell recruitment and establishing a pro‐inflammatory feedback loop that perpetuates neurovascular inflammation [218, 219]. Meanwhile, IL‐6 contributes to BBB dysfunction by activating the NF‐κB pathway in endothelial cells, thereby enhancing ICAM‐1 and VCAM‐1 expression [220, 221, 222, 223]. Additionally, increased vascular permeability and brain edema also disrupt BBB integrity [195, 217, 218, 219, 224, 225, 226, 227].

Furthermore, astrocytes also exacerbate BBB dysfunction through TGF‐β and MMP‐mediated pathways. Elevated TGF‐β isoforms in severe TBI bind to astrocytic TGFβ receptor 1, phosphorylating Smad2 to upregulate GFAP and ECM components while suppressing the tight junction protein via MMP induction [228]. MMP‐9, a key enzyme upregulated post‐TBI, degrades occludin and claudin‐5—critical transmembrane proteins that form endothelial tight junctions [217, 229, 230, 231, 232]. Moreover, parenchymal albumin extravasation stimulates astrocytic MMP release, further degrading the vascular basement membrane and amplifying BBB permeability [229]. These astrocyte‐driven mechanisms collectively impair endothelial tight junction networks, enabling neurotoxic solute influx and perpetuating neurovascular damage.

Several other interconnected astrocyte‐related mechanisms are also involved in TBI‐induced BBB dysfunction. Following TBI, bidirectional crosstalk between astrocytes and endothelial cells establishes a pathological feedback loop mediated by ET‐1. Astrocyte‐derived ET‐1 binds to endothelial ETB receptors, inducing vasogenic edema and vasospasm, while injured endothelial cells reciprocally secrete ET‐1 and vWF, activating astrocytic ETB receptors to amplify neuroinflammation [215, 216]. This reciprocal signaling enhances BBB permeability and perpetuates vascular dysfunction, whereas ETB receptor antagonism mitigates these effects [38, 119, 123, 132, 134]. What is more, the EphB3 signaling pathway is involved in BBB integrity. Astrocytes normally express ephrin‐B ligands, and after brain injury, astrocytes are activated and ephrin‐B expression is upregulated. Ephrin‐B binds to endothelial EphB3 and triggers downstream signaling involved in the negative regulation of cell survival as well as BBB maintenance. EphB3 absence promoted endothelial survival, strengthened astrocyte‐endothelial connections, and helped recover BBB functionality [233]. Besides, mitochondrial damage in astrocytes post‐TBI triggers ROS release, which directly oxidizes vascular endothelial cells, suppresses tight junction protein expression, and upregulates matrix MMPs, thereby increasing paracellular permeability [210, 234]. As mentioned above, astrocyte activation after TBI leads to glutamate accumulation, which further exacerbates BBB leakage by activating endothelial NMDA receptors, inducing calcium overload and disrupting claudin‐5 and occludin integrity [232, 235]. Additionally, astrocyte‐secreted VEGF‐A downregulates claudin‐5 and occludin expression, synergizing with TYMP to destabilize endothelial tight junctions [236, 237, 238].

In conclusion, astrocytes cause BBB damage after brain injury through multiple mechanisms, including the release of soluble factors such as MMPs, VEGF, ET‐1, and their interaction with signaling pathways such as the EphB3 pathway. These mechanisms provide potential therapeutic targets to reduce BBB damage.

### 3.4 Interactions of Astrocytes With Neurons, Oligodendrocytes, and Neural Stem Cells Post‐TBI

The post‐TBI astrocyte response also has a complex set of effects on neurons, mainly involving mechanisms such as synaptic and axonal damage and inhibition of neuronal regeneration [15]. These processes are linked to the interactions of astrocytes with various neural cells in the brain, including neurons, oligodendrocytes, and neural stem cells.

#### 3.4.1 Neuron Damage Post‐TBI Mediated by Astrocyte‐Neuron Interactions at Synapses and Axons

Neurons and astrocytes coordinate to regulate brain physiological functions through bidirectional communication. Astrocyte‐neuron interactions were initially perceived as structural, but they are now recognized as complex contributors to neurological disorders. Neurons and astrocytes share receptors and transporter proteins, enabling astrocytes to release gliotransmitters and regulate synaptic transmission by modulating neurotransmitter release/uptake in response to neuronal activity [170, 239, 240, 241, 242]. Conversely, neuronal activity also influences astrocyte behavior. For example, NMDA receptor‐mediated signaling induces astrocyte proliferation and upregulates the expression of Ptgs2, a key enzyme linked to neuroinflammation [243, 244, 245, 246, 247, 248].

When subjected to external trauma injury, neurons not only suffer damage from mechanical forces but also interact with astrocytes to evoke harmful mechanisms related to synaptic and axonal damage [168]. In this process, astrocytes lose some of their original supportive functions, such as secreting neurotrophic factors (BDNF, GDNF, etc.), which are involved in regulating synaptogenesis, neuronal differentiation, and neuronal survival [249, 250, 251, 252, 253]. In contrast, reactive astrocytes exhibit significant neurotoxic potential [254].

TBI first induces astrocyte‐specific Ephrin‐A3 overexpression, which leads to prolonged neuronal depolarization, dendritic swelling, and impaired synaptic transmission accuracy [255, 256, 257]. Second, sustained MMP‐3 upregulation disrupts synapse formation and neural signaling, compounded by MT5‐MMP‐mediated matrix degradation, ADAM‐10‐dependent synaptic protein cleavage, and dysregulated neurotrophic factors, collectively hindering functional recovery [258, 259]. Concurrently, elevated SPARC and hevin levels in reactive astrocytes amplify glutamate excitotoxicity, prolonging damage to synapses [61]. Finally, astrocytic TLR4 activation can reduce synaptic density, which is evidenced by decreased PSD‐95/VGLUT1 expression in ipsilateral cortical regions [30]. This sequence highlights the complex roles of astrocytes in post‐TBI synaptic impairment.

On the other hand, astrocytes also mediate axonal damage. It was found that the expression of CSPGs produced by astrocytes was upregulated after TBI. CSPGs are believed to primarily induce glial scar development and significantly hinder axon regeneration [260, 261, 262, 263]. CSPGs, binding to specific receptors on the axon surface, inhibit axonal migration and the expression of axon growth‐related genes [14]. For example, NgR1 and NgR3 are able to recognize CSPG. The activated co‐receptor complexes regulate cytoskeletal dynamics through Rho family GTPases, resulting in an imbalance between the polymerized state of actin fibers (as mentioned in Section 2.2) and the unpolymerized state of axon growth cones, ultimately resulting in axon collapse and hindering axon growth [264].

PTPRS, another receptor for CSPGs, may also play an inhibitory role in the process of neural regeneration [61]. Therefore, modulating the activities of these receptors is expected to promote functional recovery [265]. In addition to CSPGs, astrocytes can also inhibit axonal regeneration by upregulating the expression of the extracellular matrix protein tenascin‐C after nerve injury [266, 267].

Thus, neurons and astrocytes work together to regulate the physiological functions of the brain. When TBI occurs, astrocytes mediate synaptic damage and inhibit axonal regeneration through a variety of mechanisms. Therefore, a deep understanding of these mechanisms can help develop effective therapeutic measures to improve the prognosis of TBI patients.

#### 3.4.2 Astrocyte‐Oligodendrocyte Interactions and Myelin Damage After TBI

Oligodendrocytes predominantly produce the myelin sheaths that envelop neuronal axons, thereby facilitating rapid nerve signaling. Under physiological conditions, there are many complex interactions between astrocytes and oligodendrocytes. Astrocytes can nurture oligodendrocyte survival and specialization via secreted growth factors and nutrients. For example, BDNF promotes the proliferation and differentiation of oligodendrocyte precursor cells, which facilitates the formation and maintenance of myelin sheaths [268, 269]. Astrocytes can also create suitable survival conditions for oligodendrocytes by regulating the extracellular environment. They can take up and metabolize neurotransmitters, maintain the extracellular ionic balance, and prevent excitotoxicity from damaging oligodendrocytes [270]. In turn, oligodendrocytes are able to influence the function of astrocytes. Some signaling molecules produced by oligodendrocytes can regulate the activity of astrocytes, and the two work together to maintain the homeostasis of the nervous system [271].

Post‐TBI, demyelination activates oligodendrocyte precursor cell (OPC) proliferation and differentiation, which are essential for myelin regeneration [272], but the interactions between astrocytes and oligodendrocytes are altered, which in turn causes damage to myelin and impairs brain function. First, activated astrocytes secrete inflammatory factors, which have toxic effects on oligodendrocytes [269, 273]. Additionally, the expression of the gap junction protein connexin43 is upregulated in astrocytes, causing persistent AMPA receptor activation in OPCs, which disrupts their differentiation and maturation [274]. Finally, astrocyte‐derived exosomes modulating myelin‐related protein expression can impair myelin via TNF/FasL/glutamate secretion to induce oligodendrocyte apoptosis and reduce myelin regeneration [170, 275]. Therefore, astrocytes can cause myelin regeneration disorders by affecting the differentiation, maturation, and apoptosis of oligodendrocytes and OPCs.

In conclusion, after TBI, astrocytes and oligodendrocytes undergo a series of changes that damage myelin through complex interactions [52, 276]. Understanding these interactional pathways is crucial to developing therapies addressing myelin degradation post‐TBI.

#### 3.4.3 Effects of Astrocytes on Neural Stem Cells After TBI and Inhibition of Neural Regeneration

It has been found that the interaction of astrocytes with neural stem cells has an important impact on neural regeneration. GFAP‐deficient murine models demonstrate enhanced neurogenesis, implicating reactive astrocytes in suppressing neurogenesis [62, 277, 278, 279]. The following is an analysis of the mechanisms by which astrocytes inhibit neural regeneration through interactions with neural stem cells after TBI:

Astrocytes produce numerous factors influencing neural stem cell growth, development, and movement. Astrocytes can release substances that stimulate the expansion and development of neural stem cells, including BDNF [280], but after TBI, reactive astrocytes secrete inhibitory factors such as resistin, a pro‐inflammatory factor, which alters the neural stem cell niche to suppress neurogenesis [281]. The dysregulation of Dicer and miRNA networks in astrocytes after TBI also triggers neuroinflammation, neural degeneration, and senescence. Elevated CCL5 levels inhibit neuronal differentiation while promoting neural stem cell differentiation to astrocytes, leading to glial scar formation [74, 282]. The scar then critically impedes neuronal differentiation through both physical and chemical mechanisms, thus disrupting neural regeneration [283].

Neuroinflammation also critically inhibits neural regeneration by sustaining a pro‐inflammatory milieu through astrocytes [50]. Upon myeloid cell depletion, pro‐inflammatory astrocytes dominate cytokine production, impairing neural stem cell self‐renewal, neuronal differentiation, and survival, thereby perpetuating neurogenesis failure and functional deficits. Concurrently, reduced expression of synaptogenic factors (Gpc6, Sparcl1) in these astrocytes further limits intrinsic regenerative capacity [284, 285]. Notably, astrocyte‐derived IL‐1 exacerbates neurogenesis suppression in viral memory impairment models, underscoring a conserved inhibitory axis across CNS injuries. This could provide us with ways of investigating the mechanism of astrocyte‐mediated neural regeneration after TBI.

In summary, reactive astrocytes shift from supporting to inhibiting neurogenesis by secreting inhibitory factors, promoting glial scar formation, and sustaining neuroinflammation via dysregulated miRNA networks and pro‐inflammatory cytokines. These mechanisms suppress neural stem cell differentiation into neurons, impair self‐renewal, and create physical/chemical barriers to regeneration, highlighting astrocyte modulation as a therapeutic target for neural repair.

## 4 Signaling Pathways and Molecular Mechanisms of Astrocytes Post‐TBI

### 4.1 Activated TLR4 Signaling Pathway of Astrocyte in TBI

TLRs, a subset of PRRs, are essential for innate immunity. Although they are generally present at minimal levels in the normal brain, notable alterations in their manifestation arise post‐TBI [286, 287].

Within the intact CNS, TLR4s are primarily found in microglial cells, although this selective expression remains controversial, as it has been shown that TLR4s are also present in astrocytes and neurons cultured after injury. These TLRs are capable of detecting unique PAMPs and DAMPs [288, 289]. DAMPs activate TLRs in astrocytes [254, 290], which triggers a pro‐inflammatory response leading to neuroinflammation. For example, TLR4 activates NF‐κB through MyD88‐dependent signaling [291]. NF‐κB is a pivotal transcription factor that relocates to the nucleus upon activation and kickstarts the expression of inflammation genes [292, 293]. This mechanism triggers the discharge of numerous inflammatory substances from astrocytes, thereby intensifying the regional inflammatory reaction.

Lipopolysaccharide (LPS) is also able to activate TLR4 in astrocytes [294, 295]. TLR4/LPS interaction activates NF‐κB phosphorylation, leading to its migration into the nucleus, which in turn initiates gene expression and increases the concentrations of TNF‐α, IL‐6, IL‐1, and other cytokines. In short, LPS worsens BBB damage, cerebral swelling, and neural inflammation post‐TBI via TLR4/NF‐κB pathway activation [296, 297, 298]. Activation of TLRs also leads to enhanced expression of inflammasome components, such as NLRP3 (as mentioned in Section 3.1). Subsequent activation of the inflammasome can be triggered by potassium efflux, lysosomal disruption, and mitochondrial dysfunction. Once activated, NLRP3 promotes the maturation and release of IL‐1β and IL‐18, which further exacerbates neuroinflammation [299, 300, 301].

It is noteworthy that TLR4 activation is not an isolated event. The TLR4/MyD88 signaling axis not only directly activates NF‐κB but also activates members of the MAPK family (such as JNK and p38) via TRAF6, achieving synergistic activation of both NF‐κB and MAPK pathways (see Sections 4.2 and 4.4 for details) [302]. This “pathway convergence” amplifies the transcriptional output of inflammatory cytokines, forming the structural basis for astrocytes to achieve rapid and potent inflammatory responses.

### 4.2 Activated NF‐κB Signaling Pathway of Astrocyte in TBI

NF‐κB is a family of pleiotropic inducible transcription factors discovered in 1986. It is known to regulate innate and acquired immune functions [15, 303]. Under normal conditions, it binds to IκB in the cytoplasm and is inactive. It participates in diverse biological activities including immune response, cell proliferation, and apoptosis.

The NF‐κB transcription factor family includes five structurally conserved members: NFKB (p50), NFKB2 (p52), REL (c‐Rel), RELA (p65), and RELB. NFKB1 and NFKB2 are proteolytically processed from precursor proteins (p105 and p100, respectively), whereas REL, RELA, and RELB act directly as transcriptional activators. These five members can form various homo‐ or heterodimers that bind to specific DNA elements (κB enhancers) to mediate the transcription of target genes [304, 305].

After TBI, large amounts of inflammatory substances are released, including LPS, PAMPs, and DAMPs. They can all act as ligands to activate receptors such as TLR, IL‐1 receptor, TNF receptor, and RAGE and then activate the NF‐κB signaling pathway in astrocytes. In recognizing PAMPs and some DAMPs, TLRs interact with adaptor proteins such as MYD88 through their intracellular structural domains [291]. Similarly, IL‐1 receptor recognizes and is activated by IL1A and IL1B ligands, and also interacts with MYD88. TLRs and IL‐1 receptor assemble to form the cytoplasmic MYD88 complex, which then recruits IRAK and TAK1. Moreover, when TNF receptors are stimulated by TNFA, it recruits TRAFs, which interact with TAK1 and subsequently activate the IKK complex (including IκB kinase α/β/γ, or KK). The activated IKK complex phosphorylates IκB, an inhibitor of κB proteins. Subsequently, the phosphorylated IκB undergoes ubiquitination before being degraded by the proteasome. This process causes the liberation of NF‐κB from its cytoplasmic complex, thereby exposing its nuclear targeting signal. Typically, NF‐κB exists as a dimer, often referred to as the p50/p65 dimer. The released NF‐κB translocate to the nucleus. Notably, NF‐κB heterodimerization is a central step in the proinflammatory response of reactive astrocytes to lesion centers induced by TBI [306]. In the nucleus, NF‐κB dimers (e.g., p50/p65, p52/RelA, etc.) bind to specific DNA elements known as κB enhancers. Consequently, numerous inflammation‐related genes are activated, notably those coding for pro‐inflammatory cytokines (e.g., IL‐1A, IL‐1B, and TNFA), chemokines, and adhesion molecules. These genes enhance inflammation, significantly impacting immune cell mobilization and tissue regeneration [307, 308, 309].

In the aforementioned context, within astrocytes, NF‐κB can both transcribe proinflammatory factors to mediate neuroinflammation and upregulate ion channels/aquaporins (such as NKCC1 and AQP4, detailed in Section 2.6) to participate in edema formation. This “one pathway, multiple effects” characteristic positions NF‐κB as a central molecular hub linking diverse phenotypes of secondary injury following TBI (inflammation, edema, BBB disruption). In addition, complex cross‐regulatory interactions exist between the NF‐κB and JAK/STAT3 pathways. On one hand, NF‐κB transcriptionally upregulates cytokine expression (e.g., IL‐6), which in turn activates the JAK/STAT3 pathway via autocrine signaling [310]. On the other hand, STAT3 directly interacts with the NF‐κB p65 subunit, synergistically enhancing the transcription of inflammatory genes [311]. This “transcription factor synergy” mechanism endows the inflammatory response of astrocytes with a self‐amplifying characteristic.

### 4.3 Activated JAK/STAT Signaling Pathway of Astrocytes in TBI

JAKs belong to the cytoplasmic non‐receptor tyrosine kinase family and have a variety of downstream targets. The JAK family consists of four members: JAK1, JAK2, JAK3, and Tyk2 [13]. The JAK/STAT route mediates cytokine and growth factor signaling and is involved in a host of biological processes, including cell growth, maturation, programmed cell death, and immune regulation. The pathway consists of three major components: tyrosine kinase‐associated receptors, the JAKs, and the STAT transcription factors.

TBI triggers oxidative stress and the production of ROS, which function as signaling agents to activate the JAK/STAT cascade. In addition, DAMPs released by damaged cells, such as HMGB1, can indirectly activate the JAK/STAT pathway by binding to PRRs on astrocytes [312]. Damaged neurons, glial cells, and immune cells release various cytokines like IFN‐α, IFN‐β, and IFN‐γ, IL‐2, IL‐6, as well as CSF‐2 and CSF‐3 [15]. The various cytokines bind to the corresponding receptors to activate downstream JAK kinases. For example, IL‐2 binds to IL2Rand activates JAK1, JAK3, and Tyk2, interferons (IFNα, IFNβ, and IFNγ) bind to interferon receptors and activate JAK1 and Tyk2, whereas cytokines such as IL‐6 activate JAK1 and JAK2 [313, 314]. Upon activation, JAK kinase phosphorylates STAT transcription factors, which are intracellular transcription factors consisting mainly of STAT1‐4, STAT5a/b, and STAT6 [13].

In the signaling phase, the phosphorylated STAT is released from the receptor complex and pairs via its SH2 structural element to form a dimer [15]. Different combinations of cytokines and receptors activate specific combinations of STAT transcription factors. For example, IL2R activation results in the phosphorylation of STAT1, STAT3, and STAT5, whereas IFNR activation results in the phosphorylation of STAT1 and STAT2, and IL‐6 primarily activates STAT3. Once phosphorylated, STAT dimers translocate from the cytoplasm to the nucleus, where they bind to specific DNA sequences and interact with gene regulatory proteins to initiate the transcription of various genes. The expression products of these genes play key roles in cellular immune regulation, neuroinflammation, and other biological processes, and contribute to the inflammatory response following TBI. Studies have also shown that the JAK/STAT pathway is associated with astrocyte differentiation, glial cell activation, and glial cell gene responses after neuronal injury [315, 316]. Notably, STAT3 is considered to be a key regulator of GFAP expression during astrogenesis and astrocyte formation [23, 316, 317], which represents an important role for the JAK/STAT pathway in astrocyte activation after TBI.

### 4.4 MAPK Signaling Pathway in Astrocytes Post‐TBI

MAPKs are a family of serine/threonine protein kinases that convert extracellular signals into diverse intracellular reactions. These kinases are involved in several biological processes, including cell growth, differentiation, apoptosis, and stress responses.

The MAPK family comprises three major subfamilies: (1) extracellular signal‐regulated kinases (ERKs), including MAPK3/ERK1 and MAPK1/ERK2 (also termed p42/44 MAPK or p42/44 ERK). (2) p38 MAPKs, which consist of four isoforms (p38α, p38β, p38γ, and p38δ) encoded by distinct genes, with MAPK14/p38α being the most extensively studied. (3) JNKs, also referred to as stress‐activated protein kinases (SAPKs), such as MAPK8/JNK1 [318, 319]. Their structural and functional specificity is determined by unique upstream activators, scaffold proteins, and substrate preferences. Notably, different members of the MAPK family play distinct roles in astrocytic responses. ERK1/2 primarily responds to signals such as growth factors and ET‐1, regulating cell proliferation and cytoskeletal remodeling [320]; whereas JNK and p38 are mainly activated by inflammatory factors and oxidative stress, mediating pro‐inflammatory gene expression and apoptotic signaling [321, 322]. This functional division implies that different astrocytic responses following TBI (e.g., proliferation vs. inflammation) may be driven by distinct MAPK branches.

Post‐TBI, mechanical damage first directly disrupts cellular structures, leading to the release of intracellular signaling molecules that activate the MAPK signaling pathway [323, 324]. For example, injury‐induced membrane disruption can lead to calcium ion influx and activate calcium‐dependent proteases, which in turn stimulate initial MAPK cascade kinases [325, 326, 327]. Second, ROS also act as signaling molecules to activate upstream kinases in the MAPK pathway, such as MAP2K [328]. Third, inflammatory mediators and cytokines bind to astrocyte surface receptors, triggering activation of the MAPK signaling pathway [15]. For example, TGF‐β binds to TGF‐β receptor, IL‐1α and IL‐1β bind to IL1 receptor, TNF‐α binds to TNFR, and CXCL1 binds to CXCR2. Once a receptor is activated, signals are transmitted through upstream molecules, including MAP3K, which activates MAP2K. MAP2K, in turn, activates downstream MAPK family members, including JNK, ERK1/2, and p38 MAPK. After activation, these MAPKs enter the nucleus and regulate various transcription factors [329]. For example, JNK, ERK1/2, and p38 phosphorylate transcription factors such as ATF2 and CREB [330]. These transcription factors can act independently or together as transcription factor complexes, such as AP1 (consisting of c‐Jun and c‐Fos) and AP2ε and bind to specific DNA sequences to initiate the transcription of genes associated with immune, inflammatory, and glial markers (e.g., GFAP) [331]. Finally, albumin released from the bloodstream can also activate astrocytes through the MAPK signaling pathway. In one study, astrocyte activation by albumin triggered phosphorylation in the ERK, p38, and JNK pathways. Moreover, the presence of albumin triggered an increase of IL‐1β, nitrite, iNOS, and CX3CL1, while simultaneously reducing S100B levels, and this process ultimately kicks off the activation of the MAPK signaling pathway in astrocytes [332]. These suggest a role for the MAPK signaling pathway in mediating brain damage through astrocytes after TBI.

The MAPK pathway forms a complex signaling network with the aforementioned TLR4/NF‐κB and JAK/STAT pathways. Consequently, the astrocytic response following TBI represents the intertwined and synergistic effects of these major signaling systems: TLR/NF‐κB, JAK/STAT, and MAPK.

## 5 Discussion

The preceding sections have systematically reviewed the multifaceted roles of astrocytes in TBI pathophysiology. While significant progress has been made, several conceptual gaps and controversies warrant discussion, as they have direct implications for translational development.

The binary classification of reactive astrocytes into neurotoxic A1 and neuroprotective A2 phenotypes has provided a valuable framework, but it is increasingly recognized as an oversimplification. Single‐cell RNA sequencing studies have revealed a spectrum of transcriptional states beyond the A1/A2 binary, with many cells displaying intermediate signatures that reflect diverse microenvironmental signals [47, 57]. Moreover, the functional consequences of these phenotypes in vivo are nuanced: complement components produced by A1 astrocytes contribute to synaptic pruning, a process essential for circuit remodeling after injury, while A2‐associated factors can under certain conditions promote aberrant synapse formation or glial scar maturation [52, 250]. Future studies should move beyond simple marker‐based phenotyping and focus on functional profiling of astrocyte subsets across the spatiotemporal continuum of TBI.

A central theme emerging from this review is that key signaling pathways—TLR4/NF‐κB, JAK/STAT3, and MAPK—function as integrative hubs that transduce diverse injury signals into coordinated astrocyte responses. However, these pathways are inherently pleiotropic, and their net effect on outcome is highly context‐dependent. The JAK/STAT3 pathway exemplifies this duality: STAT3 is essential for astrocyte proliferation and glial scar formation, which contain damage in the acute phase but impede regeneration chronically [80], while its activation also drives neuropoietic cytokines that support neuronal survival [23]. Similarly, NF‐κB orchestrates both pro‐inflammatory cytokine production and the upregulation of edema‐related molecules (NKCC1, AQP4), linking inflammation and ionic dyshomeostasis [108, 114]. Yet global NF‐κB blockade in CNS injury models has yielded mixed results, sometimes impairing neuronal survival due to disruption of protective genes [303]. These findings underscore the need for cell‐type‐specific and temporally precise interventions.

Beyond canonical pathways, several emerging mechanisms warrant consideration. Astrocyte heterogeneity across brain regions remains underexplored; hippocampal and cortical astrocytes may respond differently to injury due to distinct transcriptional profiles [4]. Additionally, reactive astrocytes can acquire senescence‐associated phenotypes characterized by persistent secretion of inflammatory mediators, potentially driving chronic neurodegeneration after TBI [56]. Sex differences in astrocyte responses are also increasingly recognized and may contribute to disparities in TBI outcomes [48]. Incorporating these variables into future studies will be essential.

The conceptual framework developed here has direct therapeutic implications. Rather than pursuing broad inhibition of astrocyte reactivity, future strategies should aim to reprogram astrocyte responses in a targeted manner. Promising approaches include: targeting pathway nodes with temporal precision (e.g., inhibiting STAT3 during chronic scar formation while preserving acute neuroprotection and upregulating KLF11 expression post‐TBI can restraining inflammatory cascades) [80, 333]; modulating astrocyte‐microglia crosstalk by disrupting reciprocal amplification loops [52, 176]; harnessing A2‐promoting signals such as IL‐4 or IL‐13 to skew responses toward repair [46]; and addressing metabolic and oxidative stress through Nrf2 activators [128]. By embracing the complexity of astrocyte biology and developing strategies that modulate rather than abolish astrocyte reactivity, the field may ultimately unlock the therapeutic potential of these remarkable cells.

## 6 Conclusion

This comprehensive review positions astrocytes as central signal‐integrating hubs that orchestrate diverse secondary injury cascades following TBI. Targeting astrocyte‐specific pathways—such as modulating A1/A2 polarization or regulating ion channels—holds therapeutic potential. However, given the pleiotropic and context‐dependent nature of these pathways, future strategies should aim to reprogram astrocyte responses with temporal and cell‐type precision rather than pursuing broad inhibition. By embracing the complexity of astrocyte biology and developing strategies that modulate rather than abolish astrocyte reactivity, the field may ultimately unlock the therapeutic potential of these remarkable cells.

## Author Contributions

D.W., Y.M., and B.W. contributed equally to this work. D.W. and Y.M. drafted the article. B.W. and Y.Z. drafted the figures. J.R. and Q.S. prepared the references. Y.M., L.B., and Y.S. revised the manuscript. All authors agreed on the final draft.

## Conflicts of Interest

The authors declare no conflicts of interest.

## Data Availability

Data sharing not applicable to this article as no datasets were generated or analysed during the current study.
